# Development and characterization of novel jGCaMP8f calcium sensor variants with improved kinetics and fluorescence response range

**DOI:** 10.3389/fncel.2023.1155406

**Published:** 2023-05-18

**Authors:** Oanh Tran, Holly J. Hughes, Tom Carter, Katalin Török

**Affiliations:** Molecular and Clinical Sciences Research Institute, St George’s, University of London, London, United Kingdom

**Keywords:** biosensor, calcium, calmodulin, eNOS, fluorescence, imaging, cpEGFP

## Abstract

**Introduction:**

Genetically encoded biosensors for monitoring intracellular calcium changes have advanced our understanding of cell signaling and neuronal activity patterns in health and disease. Successful application of GCaMP biosensors to a wide range of biological questions requires that sensor properties such as brightness and dynamic range, ligand affinity and response kinetics be tuned to the specific conditions or phenomena to be investigated. Random as well as rational targeted mutations of such sensor molecules have led to a number of important breakthroughs in this field, including the calcium sensors GCaMP6f and GCaMP6f*_*u*_*. jGCaMP8f of the most recently developed generation is promising a step-change in *in vivo* imaging with further increased fluorescence dynamic range. Here, we critically examine the biophysical properties of jGCaMP8f and report development by rational design of two novel variants of jGCaMP8f.

**Methods:**

We determined the *in vitro* biophysical properties of jGCaMP8f and selected variants by fluorescence spectroscopies and compared their performance monitoring intracellular Ca^2+^ transients with previously developed fast and bright GCaMP sensors by live cell imaging.

**Results:**

We demonstrate that the physiologically highly relevant Mg^2+^ not only majorly affects the kinetic responses of GCaMPs but also their brightness and fluorescence dynamic range. We developed novel variants jGCaMP8f L27A which has threefold faster off-kinetics and jGCaMP8f F366H which shows a ∼3-fold greater dynamic range than jGCaMP8f, *in vitro* as well as in HEK293T cells and endothelial cell line HUVEC in response to ATP stimulation.

**Discussion:**

We discuss the importance of optimization of biosensors for studying neurobiology in the context of the novel variants of jGCaMP8f. The jGCaMP8f F366H variant with a large dynamic range has the potential to improve *in vivo* imaging outcomes with increased signal-to-noise ratio. The L27A variant with faster kinetics than jGCaMP8f has larger cellular responses than previous fast GCaMP variants. The jGCaMP8f generation and novel improved variants presented here will further increase the application potential of GECIs in health and disease.

## Introduction

GCaMP -style genetically encoded Ca^2+^ indicators (GECIs) superseded fluorescent Ca^2+^ indicator dyes as they enable imaging in tissues and organisms of higher integrity, with *in vivo* imaging of neuronal activity being the prize target (Dana et al., 2019; Shen et al., 2020). The development of GCaMPs stemmed from the creation of cpEGFP (Baird et al., 1999) in which the β-barrel structure of EGFP is disrupted and then corrected by Ca^2+^ binding to calmodulin (CaM) which in turn forms a high affinity complex with smooth muscle MLCK peptide (RS20). Fluorescence intensity increases as a result of a conformational change in the CaM-RS20-cpEGFP complex (Nakai et al., 2001). GCaMP generations from GCaMP to GCaMP3, GCaMP6 and GCaMP7, had increased brightness and fluorescence dynamic range (the fluorescence enhancement that occurs upon Ca^2+^ binding) (Tallini et al., 2006; Tian et al., 2009; Akerboom et al., 2012; Chen et al., 2013). The response *on*-rate determined by a conformational change in the CaM-RS20-cpEGFP complex varied from variant to variant while the response *off*-rate was generally inherently slow compared to action potential (AP) firing rate at the presynaptic terminals where GCaMPs were initially introduced (Tian et al., 2009; Steinmetz et al., 2017). Faster *off*-kinetics increase the time resolution of the observations, however, it is also important that the Ca^2+^ affinity remains appropriate for the intracellular [Ca^2+^] changes monitored so that the sensor signal represents a large fraction of the fluorescence dynamic range. Increased *off*-rates in GCaMP3*_*fast*_*, GCaMP6f*_*u*_*, and red fluorescent sensors f-RGECO1 and 2 were achieved by weakening the binding of the CaM-RS20 complex by introducing mutations to the CaM Ca^2+^-binding EF-hands and/or mutations in the RS20 peptide (Helassa et al., 2015, 2016; Kerruth et al., 2019). The targeted mutations in GCaMP3*_*fast*_*, GCaMP6f*_*u*_*, however, also lessened the restorative effect on cpEGFP and hence resulted in reduced brightness and dynamic range.

**FIGURE 1:**
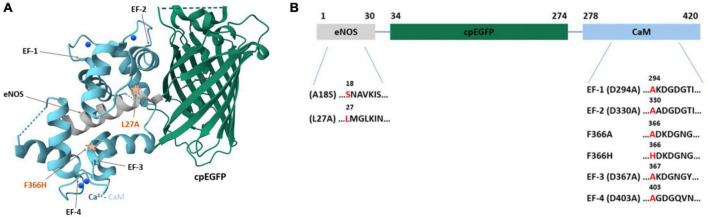
The structure of jGCaMP8f and mutation sites introduced. **(A)** The crystal structure of jGCaMP8f (PDB 7ST4). The CaM EF-hands and the position of some applied mutations is indicated by arrows. **(B)** Domain structure of jGCaMP8f with mutation sites highlighted.

jGCaMP8f (Figure 1) is a member of the latest generation of GECIs and is reported to have increased sensitivity and dynamic range (Zhang et al., 2023). jGCaMP8f also has significantly faster *off*-kinetics than GCaMP6f. We measured the biophysical properties of jGCaMP8f in order to determine how targeted mutations affect them. We report novel variants L27A and F366H with further increased *off*-rate and fluorescence dynamic range, respectively, demonstrated *in vitro* and in cellular environments.

## Materials and methods

pGP-AAV-syn-FLEX-jGCaMP8f-WPRE was purchased from Addgene (Plasmid #162379). pET30b vectors were obtained from Novagen. *E. coli* XL10-Gold and BL21 (DE3) Gold cells were purchased from Invitrogen. Subsequently, jGCaMP8f was cloned into a bacterial expression vector (jGCaMP8f pET30b) using DNA assembly (NEBuilder^®^ HiFi DNA Assembly Cloning Kit, New England Biolabs, Inc.).

### Site-directed mutagenesis

Using Q5^®^ Site-Directed Mutagenesis Kit (New England Biolabs), a range of mutations of jGCaMP8f pET30b were generated. Primers (5′-3′) used for mutagenesis were:

A18S: CAAGGAGGTGAGCACCGCCGTG
L27A: CATCGCCCGCGCAATGGGCCTGAAG
D294A (EF-1): CTCCCTATTTGCCAAGGACGGGG
D330A (EF-2): AATGAAGTAGCTGCCGACGGTG
F366A: GTTCGGTGTGGCTGATAAGGATG
F366H: GTTCGGTGTGCATGATAAGGATG
D367A (EF-3): GGTGTGTTTGCTAAGGATGGC
D403A (EF-4): AGGGAAGCAGCCATCGATGGG

After mutagenesis, DNA sequences were sent to Genewiz for checking.

### Expression and purification of jGCaMP8f and variants

jGCaMP8f and variant proteins which contained His-tag were overexpressed in *E. coli* B21 Gold cells using 0.5 mM isopropyl thio-β-D-galactoside (IPTG) then purified on a NiNTA column (QIAGEN, ÄKTA Purifier, GE Healthcare) at 4 °C as described previously (Helassa et al., 2015). Protein purity was checked using SDS-PAGE then stored at −80°C.

### Measuring protein concentrations

Protein concentration was calculated from the absorption spectra of the proteins at wavelength 280 nm using the molar extinction coefficient of 26,360 M^–1^ cm^–1^ for jGCaMP8f and all variants calculated using the ProtParam tool (Expasy).

### Fluorescence dynamic range measurements

Fluorescence emission spectra of 0.5–1 μM jGCaMP8f or variant proteins in assay buffer (50 mM HEPES-K^+^, 100 mM KCl and 5 mM EGTA in the presence or absence of 2 mM MgCl_2_, pH 7.5) at 20°C were recorded using a Fluorolog-3 spectrofluorometer (Horiba^©^) at 492 nm excitation and 500 – 550 nm emission wavelengths. Subsequently, a saturated amount of CaCl_2_ was added to obtain the maximum fluorescence emission intensity. Data were normalized and plotted using GraphPad Prism 9 software.

### Quantum yield determination

The concentration of jGCaMP8f proteins was adjusted such that the absorbance at the excitation wavelength (492 nm) was between 0.001 and 0.1. A series of dilutions was prepared in a buffered solution (50 mM HEPES, pH 7.5, 100 mM KCl, 2 mM MgCl_2_ with either 5 mM EGTA, or 1 mM CaCl_2_) and the fluorescence spectra were recorded on a Fluorolog-3 (Horiba^©^). GCaMP6f quantum yield measured in Ca^2+^ -saturated buffer was used as a reference (Φ_+*Ca*_2+ = 0.59) (Chen et al., 2013). Data were plotted as integrated fluorescence intensity as a function of absorbance and fitted to linear gradient, S. Quantum yields were obtained using the following equation: Φ_*protein*_ = Φ_*GCaMP6f*_ × (S_*protein*_/S_*GCaMP6f*_).

### pH sensitivity

To determine the apparent p*K*a for each jGCaMP8f protein, a series of buffers were prepared. Depending on their respective pH buffering range, an appropriate buffer was used for the measurements (MES for pH 6–6.5, HEPES for pH 7–8, TRIS for pH 8.5–9 and CAPS for pH 10). The pH titrations were performed by recording fluorescence spectra in Ca^2+^ -free (50 mM buffer, 100 mM KCl, 2 mM MgCl_2_, 2 mM BAPTA) or Ca^2+^-saturated (50 mM buffer, 100 mM KCl, 2 mM MgCl_2_,1 mM CaCl_2_) measuring 1 μM protein at 0.5 pH unit intervals at 20°C (Fluorolog-3, Horiba^©^). Excitation wavelength was set at 492 nm and emission in the 500–600 nm range was recorded. BAPTA was chosen as Ca^2+^ chelator because of its stable affinity for Ca^2+^ over the pH range.

### Stopped-flow fluorimetry

Ca^2+^ association and dissociation rates were measured using a TgK Scientific KinetAsyst™ double-mixing stopped-flow apparatus in single mixing mode at 20°C. The fluorescence was excited at 492 nm and the emission was collected using a 530 nm cut-off filter. For Ca^2+^ association, 500 nM jGCaMP8f or variant proteins in 50 mM HEPES-K^+^, 100 mM KCl, 2 mM MgCl_2_, 5 mM EGTA, pH 7.5 at 20°C were rapidly mixed with increasing concentrations of Ca^2+^ (CaCl_2_ in 50 mM HEPES-K^+^, 100 mM KCl, 2 mM MgCl_2_, 5 mM EGTA, pH 7.5 at 20°C), calculated free [Ca^2+^] ranged from 108 nM to 252 μM in the mixing chamber). For Ca^2+^ dissociation, 500 nM jGCaMP8f or variant proteins in 50 mM HEPES-K^+^, 100 mM KCl, 2 mM MgCl_2_, 200 μM CaCl_2_, pH 7.5 at 20°C were rapidly mixed with 50 mM HEPES-K^+^, 100 mM KCl, 2 mM MgCl_2_, 10 mM EGTA, pH 7.5 at 20°C. Experiments were repeated at least three times. Kinetic records shown are the average of at least four traces. Averaged data were fitted to a single or double exponential using KinetAsyst software (TgK Scientific) to obtain association or dissociation rates. The error quoted is the standard error of the fit. For the rate plots, the mean values obtained from at least three experiments are shown, the error bars represent S.E.M.

### Equilibrium Ca^2+^ binding

Free Ca^2+^ concentration [Ca^2+^] was calculated using the two-chelators Maxchelator program (Schoenmakers et al., 1992). Fluo-3 (Biotium) with known apparent dissociation constant for calcium (*K*_*d*_, the Ca^2+^ concentration at which half-maximum fluorescence intensity is achieved, 450 nM) was used to verify the calculated [Ca^2+^].

Fluorescence emission spectra were recorded using a Fluorolog-3 spectrofluorometer (Horiba^©^) at specific excitation and emission wavelengths, 20°C. When bound to Ca^2+^, the Fluo-3 excitation peak is at 506 nm and the emission peak is at 526 nm; the respective wavelengths for jGCaMP8f and all variant proteins are 492 nm and 512 nm. One μM jGCaMP8f or variant proteins in assay buffer (50 mM HEPES-K^+^, 100 mM KCl, 2 mM MgCl_2_ and 5 mM EGTA, pH 7.5 at 20°C) were titrated by stepwise addition of a solution of 500 mM CaCl_2_ until no further changes in fluorescence emission intensity were observed. Experiments were done in triplicates. Data was corrected for dilution, normalized and expressed as mean ± S.E.M. *K*_d_ and Hill coefficient for indication of cooperativity (*n*) were obtained by fitting the data to the Hill equation using GraphPad Prism 9 software.

### Cell culture, transfection and live cell imaging of GCaMP fluorescence in HEK293T and HUVEC cells

HEK293T cells were maintained in Dulbecco’s Modified Eagle’s Media (DMEM, high glucose, from Sigma D6546) supplemented with 10% fetal bovine serum and 4 mM L-glutamine, in 75 cm^2^ flasks. At 90% confluence, cells were grown in poly-D-lysine coated glass-bottomed dishes (MatTek Corporation) at 300,000 cells per dish for 24 h. Cryopreserved pooled primary human umbilical vein endothelial cells (HUVEC) were purchased from PromoCell GmbH (Heidelberg Germany). HUVEC were grown in a human growth medium (HGM) which comprised media M199 supplemented with 20% FCS. A total of 50 μg/mL of the antibiotic gentamicin along with ECGS (endothelial cell growth supplement; 30 μg/mL) and heparin (10 U/mL) were added to the media. HUVEC were grown on 24 well trays or 9 mm glass coverslips that had been pre-treated with porcine skin gelatine (1% w/v) for 30 min at 37°C. Cells were cultured for 4 days in a 5% CO_2_ incubator at 37 °C until fully confluent as assessed by visual examination by light microscopy.

Transfection of HEK293T was accomplished using Lipofectamine™ 2000 (Invitrogen): 2.5 μg of plasmid DNA was combined with 5 μL Lipofectamine™ 2000 reagent in Opti-MEM, this was then added to the 2 mL cell culture medium per dish. Transfection of HUVEC was by Nucleofection using an Amaxa Nucleofector 2b device and the HUVEC old transfection reagent (Lonza, Slough, UK) according to manufactures. Experiments were performed 24 h after transfection.

Transfected HEK293T cells or HUVEC were transferred to a physiological saline solution (145 mM NaCl, 5 mM KCl, 1.2 mM CaCl_2_, 1 mM MgCl_2_, 1 mM NaH_2_PO_4_, 10 mM glucose, 20 mM Na^+^-HEPES pH 7.4) and placed on the stage of an inverted Olympus IX71 fluorescence microscope. The microscope was equipped with an Olympus UPLSAPO × 100 1.40 NA objective and an OptoLED light source (Cairn Research, Faversham, UK) for EGFP (470 ± 20 nm) fluorescence. Excitation light was directed to the specimen via a 500DCXR dichroic mirror (Chroma Rockingham, USA) and emitted light collected through a 535 ± 30 nm emission filter onto an Ixon3 EMCCD camera (Andor, Belfast, UK) operated in frame transfer mode at full gain and cooled to −68 °C. Images were acquired at 20 frames/s using Winfluor software.^1^ Intracellular Ca^2+^ release in HEK293T cells was induced by ATP. ATP was administered as a bolus of 50 μL diluted in imaging buffer to give a final concentration of 50 μM in HEK293T cells and 2 μM for HUVEC. The higher sensitivity of HUVEC to ATP is reflected in the different ATP concentrations used. Full dynamic range was measured following the addition of 10 μM ionomycin. The fluorescence changes during ATP stimulation were analyzed using Winflour software. A square region of interest (ROI) was placed over a peripheral cytoplasmic region of each cell. The mean background fluorescence was measured by placing an ROI with the same dimensions at a point at which no cell was present. GraphPad Prism 9 was used to plot and fit data. Time-to-peak was determined by the time in which the rise amplitude increased from 10 to 90%. Decay *t*_1/2_ was determined from an exponential function fitted for individual records when possible, or by determining the time required for the signal dropping to half the amplitude. Data are expressed as mean ± S.E.M. For further statistical analysis one-way ANOVA tests were carried out with *post-hoc* Dunnett’s multiple comparison test. Different number of data points could be extracted for the different parameters due to the variability of individual cell responses. For example, following a clear rising phase, the decay phase may include oscillations or plateaus that complicate fitting and analysis. In some cells when ionomycin was added, no clear response was seen.

## Results

### jGCaMP8f and design of novel variants

In creating jGCaMP8f, the RS20 peptide used in most previous GCaMP designs was substituted for the CaM binding domain of nitric oxide synthase (eNOS) (Zhang et al., 2023). The relative positions of the Ca^2+^/CaM–peptide complex and cpEGFP are revealed in an adaptation of the crystal structure of jGCaMP8f (PDB ID: 7ST4) (Figure 1A).

We generated variants by introducing mutations in the CaM EF-hands and selected sites in the eNOS peptide (Figure 1B). Mutation at the EF-hands gave the D294A (EF-1), D330A (EF-2), D367A (EF-3), and D403A (EF-4) variants. In the eNOS peptide we made the A18S, L27A and in CaM the F366H and F366A mutations.

Mutations in the Ca^2+^ binding sites of CaM and/or in the eNOS peptide of jGCaMP8f will weaken the binding affinity resulting in faster calcium *off*-kinetics [L27A, A18S, D294A (EF-1), D330A (EF-2), D367A (EF-3), D403A (EF-4)] (Figure 1B). In designing the D294A (EF-1), D330A (EF-2), D367A (EF-3), and D403A (EF-4) variants, mutation of the first Asp residue of the EF-hand, known to disable Ca^2+^ binding (Jama et al., 2011), was expected to increase the Ca^2+^ *off*-rate. Likewise, the L27A and A18S mutations in the eNOS peptide were made to destabilize hydrophobic interactions with Ca^2+^/CaM with the expectation of increasing the *off*-rate, albeit to a lesser extent.

In turn, mutations that stabilize the Ca^2+^/CaM – eNOS peptide complex may increase the brightness and fluorescence dynamic range (F366A, F366H). The F366 residue in CaM is adjacent to EF-3 and is involved in hydrophobic interactions with the peptide. We designed two mutations, F366H and F366A, with the view of reducing hydrophobicity and size to increase dynamic range.

We were interested to see how these mutations affected the brightness and dynamic range, apparent Ca^2+^ affinity and kinetic responses of the variants in comparison with jGCaMP8f. We furthermore tested the response of jGCaMP8f, its novel variants and previously generated fast GCaMP variants to ATP stimulation in HEK293T cells and HUVEC.

### Biophysical characterization of jGCaMP8f and its novel variants: the effect of Mg^2+^ on the fluorescence dynamic range

**FIGURE 2:**
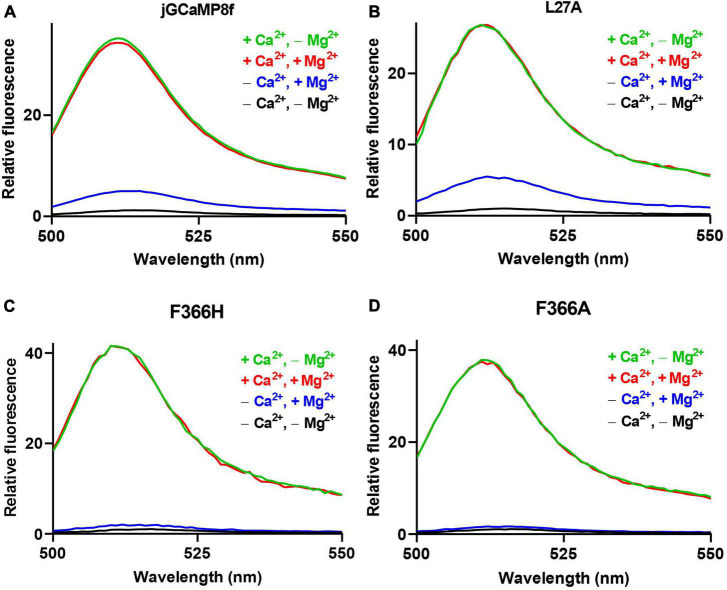
The effect of Mg^2+^ on the apo-state fluorescence intensity of jGCaMP8f and its variants. Fluorescence dynamic range measurements of **(A)** jGCaMP8f, **(B)** L27A, **(C)** F366H, and **(D)** F366A. Mg^2+^ does not affect the fluorescence intensity of the Ca^2+^-saturated form, green line (+Ca^2+^, –Mg^2+^), in 50 mM HEPES-K^+^, 100 mM KCl, 0.5 mM CaCl_2_, pH 7.5 and red line (+Ca^2+^, +Mg^2+^), in 50 mM HEPES-K^+^, 100 mM KCl, 2 mM MgCl_2_, 2 mM MgCl_2_, 0.5 mM CaCl_2_, pH 7.5. However, in the apo-form, black line (–Ca^2+^, –Mg^2+^), in 50 mM HEPES-K^+^, 100 mM KCl, 5 mM EGTA, pH 7.5, addition of 2 mM Mg^2+^ causes a fluorescence increase (–Ca^2+^, +Mg^2+^) in jGCaMP8f and its variants, thus reducing the dynamic range compared to that measured in the absence of Mg^2+^.

jGCaMP8f is reported to have a 79-fold fluorescence enhancement upon Ca^2+^ binding and an *off*-rate of 37 s^–1^ at 20°C (Zhang et al., 2023). Surprisingly, we measured an only sevenfold increase for jGCaMP8f (Figure 2A). Puzzled by the large discrepancy, we checked the experimental conditions. We found that the very high dynamic range was measured in the absence of Mg^2+^ and at pH 7.2 as opposed to our buffer that contains 2 mM Mg^2+^ and is adjusted to pH 7.5. Remeasuring the dynamic range of jGCaMP8f in the absence of Mg^2+^ (pH 7.5), we got a remarkably different result: the fluorescence of jGCaMP8f increased 31-fold upon Ca^2+^ binding. We then compared fluorescence intensities with or without added Mg^2+^ in the presence of EGTA and in the presence of Ca^2+^ (Figure 2A). We found equal intensities in Ca^2+^ with or without Mg^2+^. In the absence of Ca^2+^, however, the addition of 2 mM Mg^2+^ increased the fluorescence intensity of jGCaMP8f ∼4-fold.

A similar pattern was seen for the L27, F366H, and F366A (Figures 2B–D; Table 1). For the L27A variant only a 5.5-fold increase (comparable to the sevenfold measured for jGCaMP8f) was found in the presence of Mg^2+^, this, however, increased to 27-fold in the absence of Mg^2+^ (Figure 2B; Table 1). In the presence of Mg^2+^, Ca^2+^ binding induced 25- and 27-fold increases for the F366H and F366A variants, respectively, ∼3-fold greater than jGCaMP8f (Figures 2C, D). When measuring the dynamic range of the F366H and F366A jGCaMP8f variants in the absence of Mg^2+^ (pH 7.5), we obtained a 42-fold and a 37-fold increase upon Ca^2+^ binding, respectively, compared to 31-fold for jGCaMP8f (Figure 2; Table 1). Thus, Mg^2+^ binding to apo-jGCaMP8f and its variants significantly affects their fluorescence intensity *in vitro* and hence it is likely significant in determining the fluorescence dynamic range in resting cells. Moreover, novel variants jGCaMP8 F366H and F366A have significantly increased fluorescence dynamic range compared to jGCaMP8f (Table 1). The other mutations that were introduced had little effect on the dynamic range of jGCaMP8f (Supplementary Table 2) and thus were not investigated in detail for the effect of Mg^2+^.

**TABLE 1:** Biophysical characterisation of jGCaMP8f and novel variants.

	*K*_d_ (μM)	*n*	*F*_max_/*F*_min_ +Mg^2+^	*F*_max_/*F*_min_ −Mg^2+^	*k*_on(lim)_ (s^–1^) at 2.57 μM [Ca^2+^]	A1/A3 at 2.57 μM [Ca^2+^]	*k*_off_ (s^–1^)	*t*_1/2 off_ (ms)
jGCaMP8f	1.80 ± 0.03	2.1 ± 0.1	7.0 ± 0.1	31.1 ± 0.1	420	^a^3.5	31.5 ± 0.1	22
L27A	2.52 ± 0.02	4.6 ± 0.2	5.5 ± 0.1	26.8 ± 0.9	480	^a^5	84.4 ± 0.3	8
F366H	1.06 ± 0.03	2.3 ± 0.1	25.0 ± 0.3	41.5 ± 0.5	540	∼4	25.6 ± 0.1	27
F366A	0.90 ± 0.2	2.8 ± 0.1	27 ± 0.3	37.4 ± 0.2	445	∼4	14 ± 0.1	49

^a^See Figure 6. All measurements were carried out at pH 7.5, 20°C.

### Measurement of molecular brightness of jGCaMP8f and the L27A and F366H variants

We measured the molecular brightness of jGCaMP8f and the L27A and F366H variants, in the presence of 2 mM Mg^2+^ (Supplementary Table 1). Measurement of molar extinction coefficient values at 492 nm [ε_o(492 nm)_] in saturating Ca^2+^ of jGCaMP8f, 54,226 M^–1^cm^–1^ and a quantum yield of 0.52 gave a brightness value of 28.3 mM^–1^cm^–1^. This compares well with 29.7 mM^–1^cm^–1^ measured for GCaMP6f in the absence of Mg^2+^ (Chen et al., 2013) [N.B. in the presence of Ca^2+^, Mg^2+^ has no effect on brightness (Figure 2)]. The values measured for L27A, ε_o(492 nm)_ 48,107 M^–1^cm^–1^ (quantum yield 0.54) and F366H, ε_o(492 nm)_ 21,203 M^–1^s^–1^ (quantum yield 0.53) suggest that lower brightness of the apo-form rather than increased brightness of the Ca^2+^-bound form of the F366H variant underlies its increased fluorescence dynamic range compared to jGCaMP8f. In contrast, for the L27A variant, the Mg^2+^ induced large increase in fluorescence intensity in the apo form accounts for the loss in dynamic range. Neither fluorescence intensity nor ε_o(492 nm)_ is affected by Mg^2+^ in saturating Ca^2+^. The only available data for comparison of the apo form with and without Mg^2+^ is for jGCaMP8f (Supplementary Table 1) showing that while in Mg^2+^ ε_o(492 nm)_ is 8,413 M^–1^cm^–1^, in the absence of Mg^2+^it is reduced to 1,930 M^–1^cm^–1^, consistent with the 4-fold increase in fluorescence intensity by Mg^2+^ in the absence of Ca^2+^ (Figure 2; Table 1).

### pH dependence of fluorescence intensity and dynamic range of jGCaMP8f and the L27A and F366H variants

The pH dependence of the fluorescence intensity with or without Ca^2+^ (in the presence of 2 mM Mg^2+^) present revealed that for jGCaMP8f and the L27A and F366H variant, the intensities increased for both the Ca^2+^-bound and apo-forms, the dynamic ranges decreased with increasing pH, peaking at pH 7 (Supplementary Figure 1). p*K*_a_ values for the Ca^2+^-bound form were 7.2, 7 and 7.5, respectively.

### Kinetics of Ca^2+^ association and dissociation of jGCaMP8f: L27A, a novel variant with threefold faster off-rate

**FIGURE 3:**
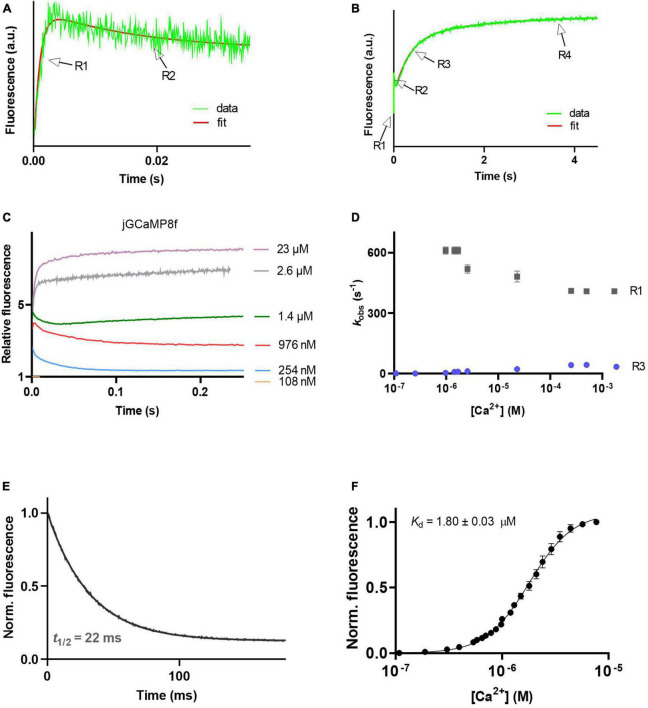
Ca^2+^ response kinetics and affinity of jGCaMP8f. **(A)** Stopped-flow association kinetic record obtained over 35 ms at 1.4 μM [Ca^2+^], the first and second phase of fluorescence changes are resolved, R1 and R2 refer to their observed rates, respectively. **(B)** Stopped-flow association kinetic record obtained over 4.5 s at 1.4 μM [Ca^2+^], revealing the four phases of fluorescence changes. Observed rates for the third and fourth phase are referred to as R1 and R2, respectively. **(C)** Association kinetic records obtained over a range of [Ca^2+^]. **(D)** Plot of observed association rates as a function of [Ca^2+^], *k*_obs_ for phases 1 (R1) and 3 (R3) are displayed. **(E)** Dissociation kinetic record and **(F)** equilibrium Ca^2+^ titration curve. The fitted parameters are shown in Table 1.

The Ca^2+^ association kinetics of jGCaMP8f measured in the presence of 2 mM Mg^2+^ at pH 7.5 proved remarkably complex, occurring in four phases. The pattern of the four phases was dependent on [Ca^2+^]. The four phases are illustrated in records taken at 1.4 μM [Ca^2+^] at a short timescale of 35 ms (Figure 3A) and a long timescale of 4.5 s (Figure 3B). The observed rates for phases 1, 2, 3, and 4 are denoted R1, R2, R3, and R4. In phases 1, 3, and 4 the fluorescence increases, however, in phase 2 the fluorescence intensity decreased. Phase 2 is no longer detected at [Ca^2+^] > 2.5 μM (Figure 3C). Phase 1 had a fast, ∼500 s^–1^ observed rate (*k*_obs_), R1 (Figure 3D). With an *off*-rate of 33.5 s^–1^, *t*_1/2_ for fluorescence decay upon Ca^2+^ sequestration was 22 ms (Figure 3E).

**FIGURE 4:**
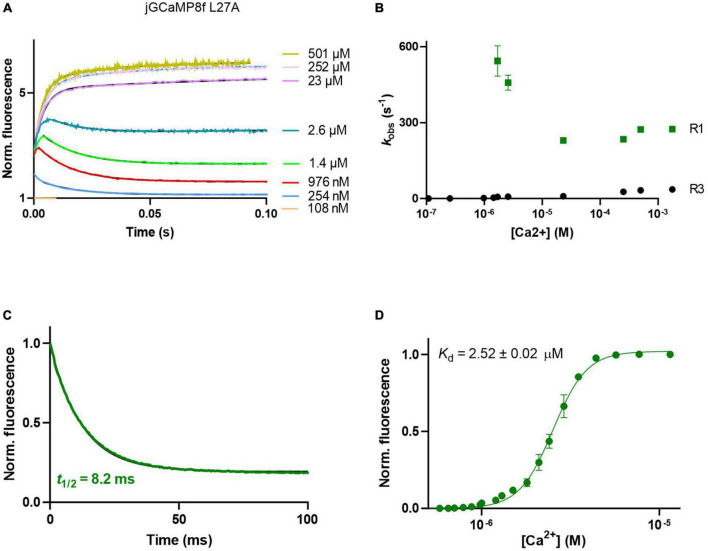
Ca^2+^ response kinetics and affinity of jGCaMP8f L27A. **(A)** Association kinetic records obtained over a range of [Ca^2+^]. **(B)** Plot of observed association rates as a function of [Ca^2+^], *k*_obs_ for phases 1 (R1) and 3 (R3) are displayed. **(C)** Dissociation kinetic record and **(D)** equilibrium Ca^2+^ titration curve. The fitted parameters are shown in Table 1.

The L27A variant had similarly complex on-kinetics to jGCaMP8f (Figure 4A), with fast (∼300 s^–1^) initial *on*-rate (R1) (Figure 4B). The *off*-rate of the L27A variant was 84.4 s^–1^ (decay *t*_1/2_ of 8.2 ms) (Figure 4C) threefold faster than that of jGCaMP8f.

**FIGURE 5:**
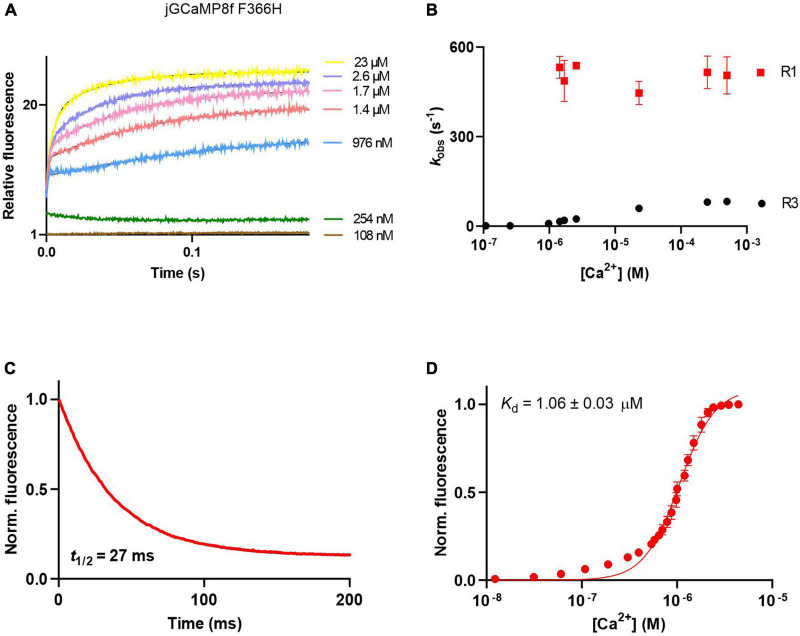
Ca^2+^ response kinetics and affinity of jGCaMP8f F366H. **(A)** Association kinetic records obtained over a range of [Ca^2+^]. **(B)** Plot of observed association rates as a function of [Ca^2+^], *k*_obs_ for phases 1 (R1) and 3 (R3) are displayed. **(C)** Dissociation kinetic record and **(D)** equilibrium Ca^2+^ titration curve. The fitted parameters are shown in Table 1.

jGCaMP8f F366H showed a similar pattern of association kinetics to jGCaMP8f and the L27A variant (Figure 5A), with > 500 s^–1^ initial *on*-rates (R1) measurable in the μM [Ca^2+^] range (Figure 5B). The F366H variant had a similar *off*-rate of 33 s^–1^ to jGCaMP8f (Figure 5C). The F366A variant also had improved fluorescence enhancement (27-fold), however, both the *on*- and the *off*-rates of this variant are slowed down to 171 s^–1^ and 14 s^–1^, respectively (Supplementary Figures 3G, H; Table 1).

Supplementary Figure 3 shows the *on*- and *off*-kinetic responses of the other variants. Notably, the D367A (EF-3) and D403A (EF-4) mutations had very little effect on either the kinetic or fluorescence responses of jGCaMP8f (Supplementary Figures 3I, J; Supplementary Table 1). The A18S variant had a slightly increased, eightfold dynamic range and slightly slower *off*-rate (25 s^–1^) than jGCaMP8f (Supplementary Table 2).

Measurement of the Ca^2+^ *on*- and *off*-response rates of GCaMPs informs on the interaction mechanisms. Furthermore, simple rate and amplitude measurements are usually sufficient as screening tools for judging the sensors’ potential for cell imaging. However, given the complexity of their *on*-kinetics, for jGCaMP8f and its variants detailed analysis of the rate and amplitude patterns of the four phases of the *on*-response was required to see if the [Ca^2+^] range where the fast *on*-phase is the most significant is compatible for intracellular applications. Such analysis helped predict if our novel variants perform well in the cellular environment. As examples, the rate and amplitude patterns of the four phases of the *on*-response of jGCaMP8f, the L27A, D294A (EF-1), D330A (EF-2), and F366H variants were studied.

**FIGURE 6:**
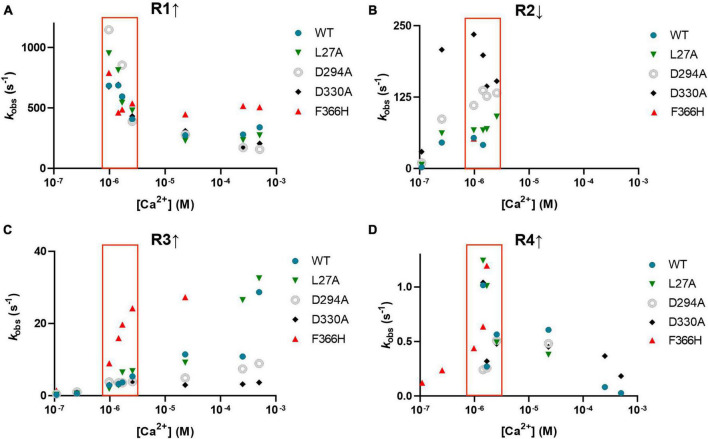
Observed rates of each of the four phases of the association kinetic records measured over a range of [Ca^2+^] of jGCaMP8f and selected variants. **(A)** R1 ↑ **(B)** R2 ↓, **(C)** R3 ↑ and **(D)** R4 ↑. The low μM [Ca^2+^] range, in which the response rates and patterns are considered most relevant to function in the cellular environment, is highlighted by a red box.

Figure 6 shows the Ca^2+^ dependence of the four phases of the *on*-fluorescence response of jGCaMP8f and its L27A, D294A (EF-1), D330A (EF-2), and F366H variants, revealing that rates (R1 ↑) of the fast, rising phase (400–1,000 s^–1^) are highest at around 1–2.5 μM [Ca^2+^] for all five proteins (Figure 6A). In the second phase R2 ↓ of 50–250 s^–1^, the fluorescence change is inverted and shows little Ca^2+^ dependence (Figure 6B). The third phase is upward with a slow *k*_obs_ (R3 ↑) of 5 s^–1^ which increases to ∼30 s^–1^ in the 1–2.5 μM [Ca^2+^] range for the F366H variant, while only at high [Ca^2+^] for the other four proteins (Figure 6C). *k*_obs_ for the fourth phase (R4 ↑) is slow ∼1 s^–1^ (Figure 6D). The low μM [Ca^2+^] range, in which the response rates and patterns are considered most relevant to function in the cellular environment, is highlighted by a red box.

**FIGURE 7:**
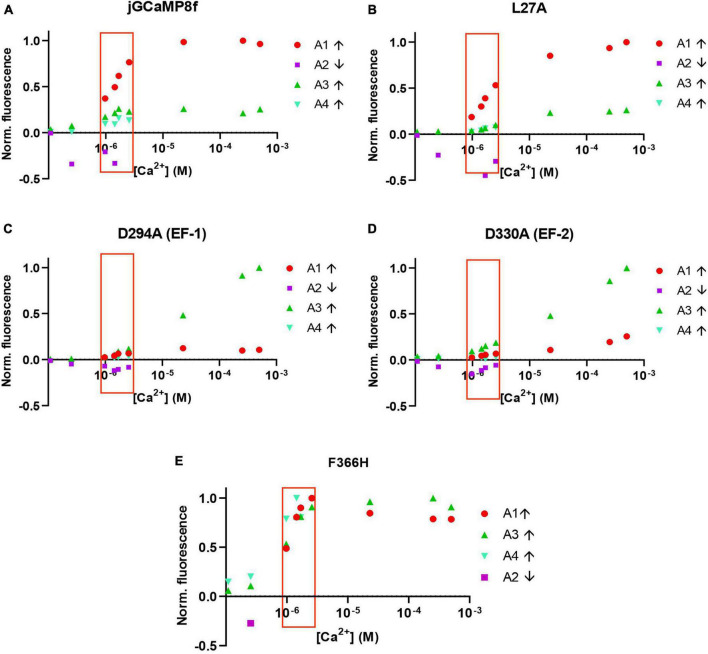
Normalized amplitudes of each of the four phases of the association kinetic records measured over a range of [Ca^2+^] of jGCaMP8f and selected variants. **(A)** jGCaMP8f, **(B)** L27A, **(C)** D294A (EF-1), **(D)** D330A (EF-2), and **(E)** F366H. The low μM [Ca^2+^] range, in which the response rates and patterns are considered most relevant to function in the cellular environment, is highlighted by a red box.

Analysis of the amplitudes (A1, A2, A3, and A4, corresponding to phases 1, 2, 3, and 4 with rates R1, R2, R3, and R4) shows that for the jGCaMP8f, the L27A and F366H variants the rapid first phase represents the major fraction of the amplitude (Figures 7A, B, E). In contrast, for the D294A (EF-1) and D330A (EF-2) variants the major part of the amplitude derives from the slow third phase, but even that only becomes significant at [Ca^2+^] of 20 μM and greater, with time to peak of ∼200 ms (Figures 7C, D). Therefore, even though, the *off*-rates for these two variants are quite fast (153 and 303 s^–1^, Supplementary Figures 3C–F; Supplementary Table 2), they would only function as useful sensors at [Ca^2+^] concentrations that exist in organelles rather than the cytoplasm and only in time-insensitive experimental setups. The F366H variant scores best in terms of reaching maximum amplitude in the first, fastest phase (incidentally, also in phase 3 and 4 (Figure 7E). The low μM [Ca^2+^] range, in which the response rates and patterns are considered most relevant to function in the cellular environment, is highlighted by a red box.

### Equilibrium Ca^2+^ binding

jGCaMP8f and all its variants had apparent *K*_d_ for Ca^2+^ in the low 1–4 μM range with Hill coefficient (*n*) values of 2–5 (Figures 3F, 4D, 5D; Supplementary Figure 2; Table 1; Supplementary Table 2). In our hands, the apparent *K*_d_ of jGCaMP8f for Ca^2+^ was 1.80 ± 0.03 μM (Figure 3F), in contrast with a *K*_d_ of 334 ± 18 nM measured in the absence of Mg^2+^ (Zhang et al., 2023). The Hill coefficient for Ca^2+^ binding was 2.1 ± 0.1–0.2 in both sets of measurements (Table 1). For the L27A variant a *K*_d_ for Ca^2+^ of 2.52 ± 0.02 μM with *n* of 4.6 ± 0.2 was measured (Figure 4D; Table 1). The F366H and F366A variants had *K*_d_ for Ca^2+^ of 1.06 ± 0.03 μM and 0.90 ± 0.2 μM, respectively, with *n* of 2.3 ± 0.1 and 2.8 ± 0.1, respectively (Figure 5D; Table 1; Supplementary Figure 2).

### Monitoring intracellular Ca^2+^ elevation by GCaMP Ca^2+^ sensors

**FIGURE 8:**
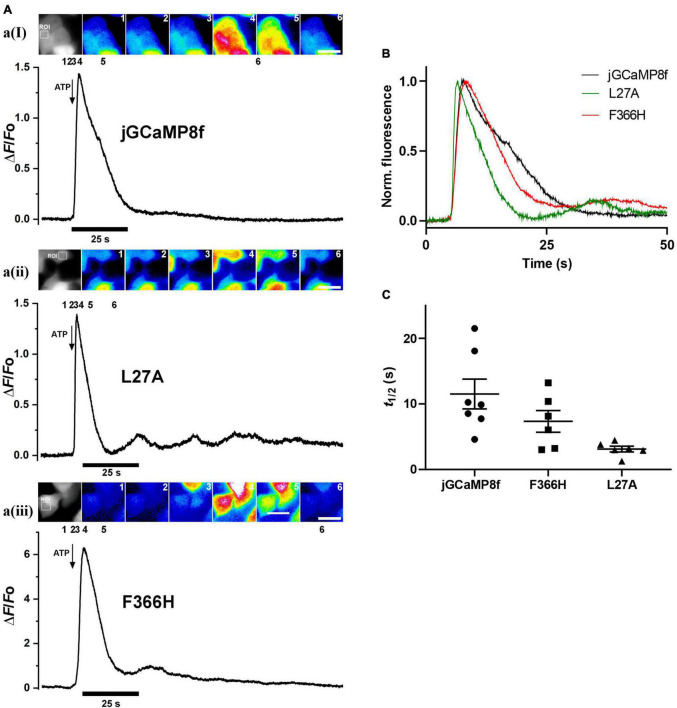
Fluorescence live cell imaging of jGCaMP8f and its L27A and F366H variants in HEK293T cells at 37°C. **(A)** Image montages and Δ*F*/*F*_o_ plots of **(ai)** jGCaMP8f, **(aii)** jGCaMP8f L27A, and **(aiii)** jGCaMP8f F366H transfected HEK293T cells with the region of interest (ROI) shown in the grayscale image. The time points where the selected frames originate from are indicated by numbers 1–6. The scale bar represents 20 μM. Addition of 50 μM ATP is indicated by the arrows. **(B)** Normalized records and **(C)** dot plot of the decay *t*_1/2_ values for jGCaMP8f and its L27A and F366H variants, displaying the individual data points and S.E.M.

From *in vitro* experiments, compared with jGCaMP8f, L27A has faster kinetics and F366H has greater dynamic range whilst kinetics is preserved therefore these variants were tested in cells at 37°C. [Ca^2+^] elevation was induced in HEK293T cells by stimulation with 50 μM ATP (Supplementary Videos 1–3). Figure 8A shows a montage of images at the indicated points of the Δ*F*/*F*_o_ time course for each jGCaMP8f, the L27A and F366H variants at the indicated region of interest. In each case, the first Ca^2+^ transient is followed by one or more smaller further Ca^2+^ rises, indicating oscillating [Ca^2+^]. This can often be seen in response to ATP stimulation. jGCaMP8f detected intracellular Ca^2+^ elevation with a fluorescence increase, Δ*F*/*F*_o_ of 2.4 (Δ*F*/*F*_o_ measured in response to 2 μM ionomycin was 3.2). The F366H variant responded with a Δ*F*/*F*_o_ of 9 (Δ*F*/*F*_o_ measured in response to 2 μM ionomycin was 12), while for L27A the Δ*F*/*F*_o_ was 1 (Δ*F*/*F*_o_ measured in response to 2 μM ionomycin was 2.8). Time to peak values were 1.4 s, 1.8 s and 1 s for jGCaMP8f and its F366H and L27A variants, respectively. Thus, the *on*-kinetic responses of the three proteins were similar, however, the decay *t*_1/2_ for the L27A variant of 4.6 s was 2.3-fold smaller than for jGCaMP8f (10 s) and F366H variant (11 s). Moreover, the amplitude of fluorescence increase for jGCaMP8f F366H was 3.75-fold greater than for jGCaMP8f (Figure 8; Table 2), while preserving the fast off-kinetics of jGCaMP8f.

**TABLE 2:** Live cell imaging of ATP evoked intracellular Ca^2+^ transients monitored by GCaMP variant sensors in HEK293T cells.

	jGCaMP8f	jGCaMP8f L27A	jGCaMP8f F366H
Δ*F*/*F*_o_ (ATP) HEK293T	2.4 ± 0.4	1.0 ± 0.1	9.0 ± 0.9
Δ*F*/*F*_o_ ionomycin HEK293T	3.2 ± 0.2	2.8 ± 0.2	11.8 ± 1.1
Time to peak (s) (ATP) HEK293T	1.4 ± 0.3	1.0 ± 0.1	1.8 ± 0.5
*t*_1/2 (decay)_ (s) (ATP) HEK293T	11.5 ± 4.7	3.1 ± 1.1	7.3 ± 4.0

**FIGURE 9:**
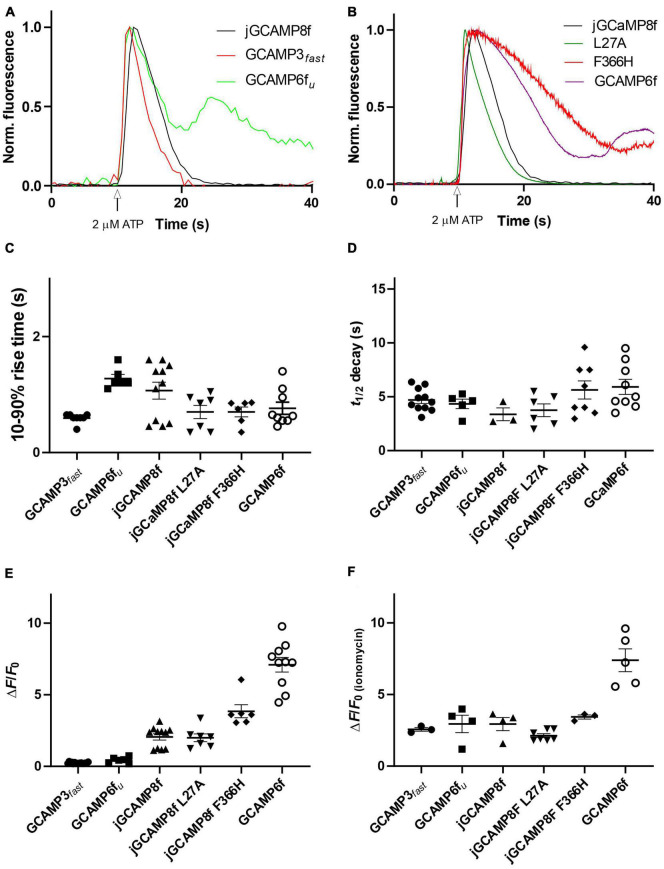
Fluorescence live cell imaging of jGCaMP8f, GCaMP6f*_*u*_* and GCaMP3*_*fast*_* in HUVEC at 37°C. **(A)** Example records. Ca^2+^ transients evoked by 2 μM ATP monitored by jGCaMP8f, GCaMP6f*_*u*_*, and GCaMP3*_*fast*_*. Normalized data are shown. **(B)** Example records. Ca^2+^ transients evoked by 2 μM ATP monitored by jGCaMP8f [duplicate of record shown in panel **(A)**], jGCaMP8f L27A, jGCaMP8f F366H, and GCaMP6f. Normalized data are shown. Dot plots of **(C)** 10–90% rise time (s), **(D)** decay *t*_1/2_ (s), **(E)** Δ*F*/*F*_o_ in response to 2 μM ATP and **(F)** after adding 10 μM ionomycin. Dot plots show individual data points and thus provide the n numbers (number of cells) for the experiments. Mean and S.E.M. is indicated.

jGCaMP8f has 13-fold faster decay rate than GCaMP6f (*in vitro* decay *t*_1/2_ 22 ms at 20°C). Previously, GCaMP3*_*fast*_* and GCaMP6f*_*u*_* were developed which had *in vitro* decay *t*_1/2_ of 11 and 8 ms, respectively, at 20°C (Helassa et al., 2015, 2016). We were interested to see how these differences in *in vitro off*-rates translate to differences in cell responses at 37°C. Therefore, we compared the kinetics of the responses of jGCaMP8f and three previously developed fast decay sensors GCaMP3*_*fast*_* and GCaMP6f*_*u*_* and GCaMP6f, in addition to the two novel jGCaMP8f variants, L27A and F366H, to 2 μM ATP in HUVEC (Figures 9A, B). As seen in the record for GCaMP6f*_*u*_*, the first Ca^2+^ transient is followed by a smaller second Ca^2+^ rise, indicating oscillating [Ca^2+^]. This can often be seen in response to ATP stimulation and can distort the response kinetics. Comparison of the rise times revealed no significant difference between the sensors when compared to jGCaMP8f except for GCaMP3*_*fast*_* the rise time for which was significantly shorter than for the others, with a *p*-value of 0.1 (Figure 9C). The decay times of the responses suggest similar kinetics for the sensors, however, accurate comparison was hindered by the saturation effect most noticeable for GCaMP6f and the jGCaMP8f F366H variant (Figures 9B, D; Table 3). In terms of response dynamic ranges, jGCaMP8f sensitively detected intracellular Ca^2+^ elevation with a fluorescence increase, Δ*F*/*F*_o_ of 2.1, similar to the 2.4 measured in HEK293T cells and significantly higher than 0.23 (*p* > 0.0001) for GCaMP3*_*fast*_* and 0.44 (*p* = 0.003) for GCaMP6f*_*u*_* (Figure 9E; Table 3). There was no significant difference between jGCaMP8f and its L27A variant, however, both the jGCaMP8f F366H variant and GCaMP6f responded with 1.9- (*p* = 0.0009) and 3.5-fold (*p* < 0.0001) greater Δ*F*/*F*_o_ than jGCaMP8f. The full dynamic ranges (Δ*F*/*F*_o_ measured in response to 10 μM ionomycin) of the sensors were similar with, interestingly, GCaMP6f exceeding even that of jGCaMP8f F366H (*p* = 0.002) (Figure 9F; Table 3), suggesting that a lower apparent *K*_d_ for Ca^2+^ compared to that of GCaMP3*_*fast*_* and GCaMP6f*_*u*_* is likely to underlie the much greater amplitude of jGCaMP8f in response to 2 μM ATP.

**TABLE 3:** Live cell imaging of ATP evoked intracellular Ca^2+^ transients monitored by GCaMP variant sensors in HUVEC.

	jGCaMP8f	GCaMP3*_*fast*_*	GCaMP6*_*fu*_*	jGCaMP8f L27A	jGCaMP8f F366H	GCaMP6f
Δ*F*/*F*_o_ ATP HUVEC	2.1 ± 0.2	0.23 ± 0.01	0.44 ± 0.08	2.0 ± 0.3	3.85 ± 0.45	7.1 ± 0.5
Δ*F*/*F*_o_ ionomycin HUVEC	2.9 ± 0.5	2.6 ± 0.1	3.0 ± 0.6	2.1 ± 0.1	3.4 ± 0.1	^a^5.8
Time to peak (s) (ATP) HUVEC	1.1 ± 0.1	0.59 ± 0.03	1.3 ± 0.1	0.7 ± 0.1	0.7 ± 0.1	0.8 ± 0.1
*t*_1/2 (decay)_ (s) (ATP) HUVEC	3.4 ± 0.6	4.7 ± 0.3	4.3 ± 0.4	3.7 ± 0.6	5.6 ± 0.6	5.9 ± 0.7

^a^Single value obtained, other cells were not responsive to ionomycin.

## Discussion

As Ca^2+^ binding to CaM and hence its interaction with target peptides and proteins is affected by Mg^2+^ and Mg^2+^ is also present in the cells, our measurements are carried out in a buffer containing 2 mM MgCl_2_ and found striking differences in the dynamic ranges when comparing results obtained in the absence of Mg^2+^. jGCaMP8f is reported to have a significantly greater fluorescence dynamic range than the GCaMP6 generation, in comparable conditions, in the absence of Mg^2+^ at pH 7.2 (Zhang et al., 2023). Brightness measurements reveal similar brightness (Supplementary Table 1) and, in Mg^2+^, greater dynamic range of 14.3 for GCaMP6f (Helassa et al., 2016) than 7, measured for jGCaMP8f. The lower pH of 7.2 compared to our 7.5 used could have further contributed to the very high dynamic range value measured in the absence of Mg^2+^ as jGCaMP8f shows increasing dynamic range as pH is lowered (Supplementary Figure 1). The experimental conditions may explain the differences in the measured *K*_*d*_ values, too.

Ca^2+^ binding increases the quantum yield of the C-lobe Tyr fluorescence. Mg^2+^ has the same effect, though to a lesser degree (Kilhoffer et al., 1981). Thus, although Mg^2+^ binding to N-lobe EF-hands stabilizes the closed state (Senguen and Grabarek, 2012), its binding to the C-lobe resembles that of Ca^2+^. Therefore, in the absence of or at low, resting level [Ca^2+^], Mg^2+^ that is present in cells will bind to CaM and affect the interaction of the CaM C-lobe with the target peptide or protein. Thus, it is not surprising that in the presence of 2 mM Mg^2+^ the basal fluorescence of jGCaMP8f and its variants is increased, interestingly, to different extents. Most pronounced was the effect of Mg^2+^ on the L27A variant, increasing fluorescence intensity of the apo-state sixfold. Thus, reducing its fluorescence dynamic range to 5.5 even though the brightness of 25.9 mM^–1^cm^–1^ for the L27A variant is comparable to that of jGCaMP8f. A lesser effect by Mg^2+^ is seen on the apo-state for the F366H and F366A variants with a 2- and 1.6-fold increase, respectively. This property is highly relevant in the application of GCaMP-type Ca^2+^ sensors, as indeed for other CaM-based sensors, e.g., NCaMP7 (Subach et al., 2020). Mg^2+^ will furthermore affect the apparent *K*_d_ for Ca^2+^ and the kinetics of Ca^2+^ sensing and the Ca^2+^ *on*-kinetics of jGCaMP8f could be affected by the release kinetics of Mg^2+^ from Mg^2+^-bound jGCaMP8f.

The complex *on*-kinetics of jGCaMP8f and its variants at [Ca^2+^] < 2.5 μM provides an insight into the Ca^2+^ binding mechanism. The very fast initial on-rate (R1) of ≥500 s^–1^ likely represents Ca^2+^ binding to the CaM EF-1 and EF-2 sites of the N-lobe, the transient drop of fluorescence in the second phase (R2) then corresponds to Ca^2+^dissociation from the N-lobe as the [Ca^2+^] is not sufficient for saturating N-lobe Ca^2+^ binding. Slow Ca^2+^ binding to the C-lobe follows in phase 3 (R3) and the binding is stabilized in phase 4 (R4). At [Ca^2+^] > 2.5 μM, the N-lobe can hold onto Ca^2+^ better and the second phase is no longer detectable. Thus, fluorescence rapidly increases in a more straightforward manner. Our data with the D294A (EF-1) and D330A) (EF-2) variants, which only show the slow 3*^rd^* and 4*^th^* phases, support this explanation.

The pattern of *on*-rates and amplitudes is favorable for the L27A and F366H variants with fast initial on-rates (R1) of ≥500 s^–1^ in the physiologically relevant 1–2.5 μM intracellular [Ca^2+^], together with jGCaMP8f. The *on*-rate of jGCaMP8f and its L27A and F366H variants is 3.4 and 1.5-fold faster than that the fast decay GCaMP3*_*fast*_* and GCaMP6f*_*u*_*, respectively, at 20°C (Helassa et al., 2015, 2016).

jGCaMP8f and the F366H variant have a 13-fold faster *off*-rate than GCaMP6f with decay *t*_1/2_ of 22 ms. The jGCaMP8f L27A variant is a further 3∼fold faster with decay *t*_1/2_ of 8 ms at 20°C, similar to GCaMP3f*_*fast*_* and GCaMP6f*_*u*_* (Helassa et al., 2015, 2016). Notably, the *off*-rate increase of GCaMPf*_*fast*_* and GCaMP6f*_*u*_* was achieved by the combination of mutating the CaM EF-hand 3 and a hydrophobic residue (W) involved in peptide binding to the CaM C-lobe. In contrast, in jGCaMP8f EF-3 or the EF-4 mutation that disabled Ca^2+^ binding did not significantly affect the properties of jGCaMP8f, while mutating EF-1 and EF-2 resulted in very fast *off*-rates but drastically slowed-down *on*-response times, implicating their important role in binding the eNOS peptide which is essential for restoring fluorescence of the cp-EGFP β barrel.

The fluorescence dynamic range of ∼5 is similar for the fast-decay jGCaMP8f L27A, GCaMPf*_*fast*_* and GCaMP6f*_*u*_* variants, in each case reduced compared to the parent GCaMP. In contrast, jGCaMP8f F366H shows a 25-fold fluorescence enhancement upon Ca^2+^ binding, 3-fold greater than jGCaMP8f in our hands (in the presence of Mg^2+^), but similar *off*-rate to jGCaMP8f. Thus, simultaneously increasing brightness, dynamic range and *off*-rate of GCaMPs remains elusive.

The greater amplitude (3.75-fold in HEK293T and 1.9-fold in HUVEC) of the response to ATP stimulation by our F366H variant compared to jGCaMP8f is consistent with the 3-fold difference we measured *in vitro*. However, response to cell [Ca^2+^] transients is not always entirely consistent with what the *in vitro* experiments predict. There are many possible reasons for this, for example there can be affinity differences in the cells compared to what is measured in solution, pH changes can affect sensor responses.

Comparison of jGCaMP8f, its L27A and F366H variants in two different cell lines reveals differences in sensor responses. This is not surprising as cell responses are heterogenous, different cell lines respond differently to hormones, e.g., due to the purinergic receptor subtypes present and subtleties in signal transduction ATP action on individual cells often results in Ca^2+^ transients of different magnitude and time course. Our cell data show the expected range of response times and amplitudes, similar to other reports of GCaMP cell responses (Zhang et al., 2023). Interestingly, while our data in HEK293T cells clearly show a faster decay time for L27A and a larger dynamic range for F366H than jGCaMP8f, the differences are less clear cut in HUVEC. The decay times of the responses suggest similar kinetics for the sensors, however, accurate comparison was hindered by the saturation effect most noticeable for GCaMP6f and the jGCaMP8f F366H variant. The dynamic range for F366H is larger than either of the other two variants, however, it is diminished in HUVEC compared to HEK293T cells. In conclusion, sensor responses differ both quantitatively and qualitatively in different cell types and experimental conditions, which is partly expected. Hormone, e.g., ATP-induced intracellular transients are particularly large in endothelial cells, e.g., HUVEC, reaching 10–30 μM (Carter and Ogden, 1997) which is likely the reason for signal saturation, which is evident from the non-exponential decay kinetics of GCaMP6f and the jGCaMP8f F366H variant. Ionomycin in HUVEC only increases the dynamic range for GCaMP3*_*fast*_* and GCaMP6f*_*u*_*, both these indicators have higher *K*_*d*_-s than the other featured GCaMPs.

We note that in spite of the large Ca^2+^ transient in HUVEC (Carter and Ogden, 1997) compared to HEK293T cells, the sensor dynamic ranges are diminished, except for GCaMP6f. This may be attributable to less transparency due to the geometry and composition of HUVEC. The apparent *K*_d_ values of cpEGFP-based sensors can also be affected by the cell composition, e.g., glutamate sensors have much higher affinity following cell expression.

In summary, we present a thorough characterization of jGCaMP8f and two newly developed variants, the faster decay L27A and F366H with an increased dynamic range, through which we highlight the complexities of the kinetics of this sensor generation. We demonstrate how Mg^2+^, present at mM concentrations in all cells, increases the apo-state fluorescence and is thus an important factor in defining the achievable changes in fluorescence dynamic range of GCaMP-type Ca^2+^ sensors in physiological conditions. We demonstrate the superior performance of two novel variants of jGCaMP8f, one with faster kinetics and one with increased fluorescence dynamic range (in the presence of Mg^2+^), both *in vitro* and in cellular environments. The *off*-kinetics of L27A is as fast as that of GCaMP3*_*fast*_* and GCaMP6f*_*u*_*, however, the jGCaMP8f L27A variant may be more advantageous in imaging applications as it has a greater fluorescence dynamic range in the cellular environment than the earlier fast variants. The jGCaMP8f F366H variant gives a 2–3.75-fold greater fluorescence increase in cells than jGCaMP8f whilst preserving its fast kinetics unless saturation distorts its response kinetics.

Genetically encoded Ca^2+^ indicators are highly applicable for studying cell signaling involving transient rises of intracellular [Ca^2+^] to understand physiological or disease conditions. An important consideration for the application for GECIs for imaging neural activity is the kinetics of the intracellular [Ca^2+^] transient generated by AP-evoked Ca^2+^ influx and terminated by Ca^2+^ pumps, which is slower than the ion channel activities driving APs. In cells, the millisecond response times of GECIs translate into transients over seconds. Nevertheless, increasing the *off*-rate of GCaMPs is important for faithful reporting of intracellular Ca^2+^ transients and for increasing the resolution of oscillating Ca^2+^ transients. However, there are still limitations to the sensitivity and fidelity of detection. It is important to understand how closely the fluorescence changes map the neural activity under investigation. We note that many *in vivo* or in cell studies cannot give detailed kinetic data of Ca^2+^ signals. This may be explained by the low expression level and/or low brightness of GECIs *in vivo*, their action as a Ca^2+^ buffer, slow kinetics, the complexity arising from the non-linearity of the data (for a review, see Rose et al., 2014). jGCaMP8f of the latest GECI generation has showed pronounced results in repetitive stimulation and monitoring Ca^2+^ transients in larval Drosophila and mouse visual cortex models (Zhang et al., 2023). Our jGCaMP8f variants L27A and F366H offer further advantages in kinetics and dynamic range in the cellular environment (in the presence of Mg^2+^), respectively, therefore have potential applications to study physiological or disease conditions. For example, simultaneous recording of APs and GCaMP6s or GCaMP6f fluorescence *in vivo* in the primary visual cortex of transgenic mice revealed that in typical population imaging conditions only ∼10% of single AP events were detected (Huang et al., 2021). jGCaMP8f F366H with a large dynamic range has the potential to improve this outcome by increasing the signal-to-noise ratio. Conversely, a study in Huntington’s disease mouse models using GCaMP3 revealed the increase of evoked Ca^2+^ signals, but a significant reduction in the frequency, duration, and amplitude of spontaneous Ca^2+^ signals (Jiang et al., 2016). jGCaMP8f L27A with faster kinetics and larger cellular responses than previous fast GCaMP variants might help understand the underlying mechanisms of dysfunctional Ca^2+^ signaling.

Genetically encoded Ca^2+^ indicators are important and powerful tools in the investigation of neuro- and other pathologies. The latest jGCaMP8f generation and novel improved variants presented here will further increase the application potential of GECIs in health and disease.

## Data availability statement

The original contributions presented in this study are included in the article/Supplementary material, further inquiries can be directed to the corresponding author.

## Author contributions

OT generated the D294A (EF-1), D330A (EF-2), A18S as well as designed, generated the F366A and F366H variants by SDM, and carried out all *in vitro* experiments including expression, purification, and measurements. HH and TC carried out the live cell imaging. KT designed the project. KT and OT wrote the manuscript. All authors contributed to the article and approved the submitted version.

## Funding

This work was funded by BBSRC grant BB/M02556X/1 to KT.

## Footnotes

1
http://spider.science.strath.ac.uk
