# Supplementary material for: Development and characterization of novel jGCaMP8f calcium sensor variants with improved kinetics and fluorescence response range
Source: Front Cell Neurosci. 2023 May 18;17:1155406. doi: 10.3389/fncel.2023.1155406 (PMC10234427; doi:10.3389/fncel.2023.1155406)
Supplement: Supplementary file 1 [file Table_1.DOCX]

Supplementary Material

Development and optimisation of genetically encoded calcium sensors

Oanh Tran^1^, Holly J. Hughes^1^, Tom Carter^1^, Katalin Török^1*^

*** Correspondence:** Katalin Török (k.torok@sgul.ac.uk)


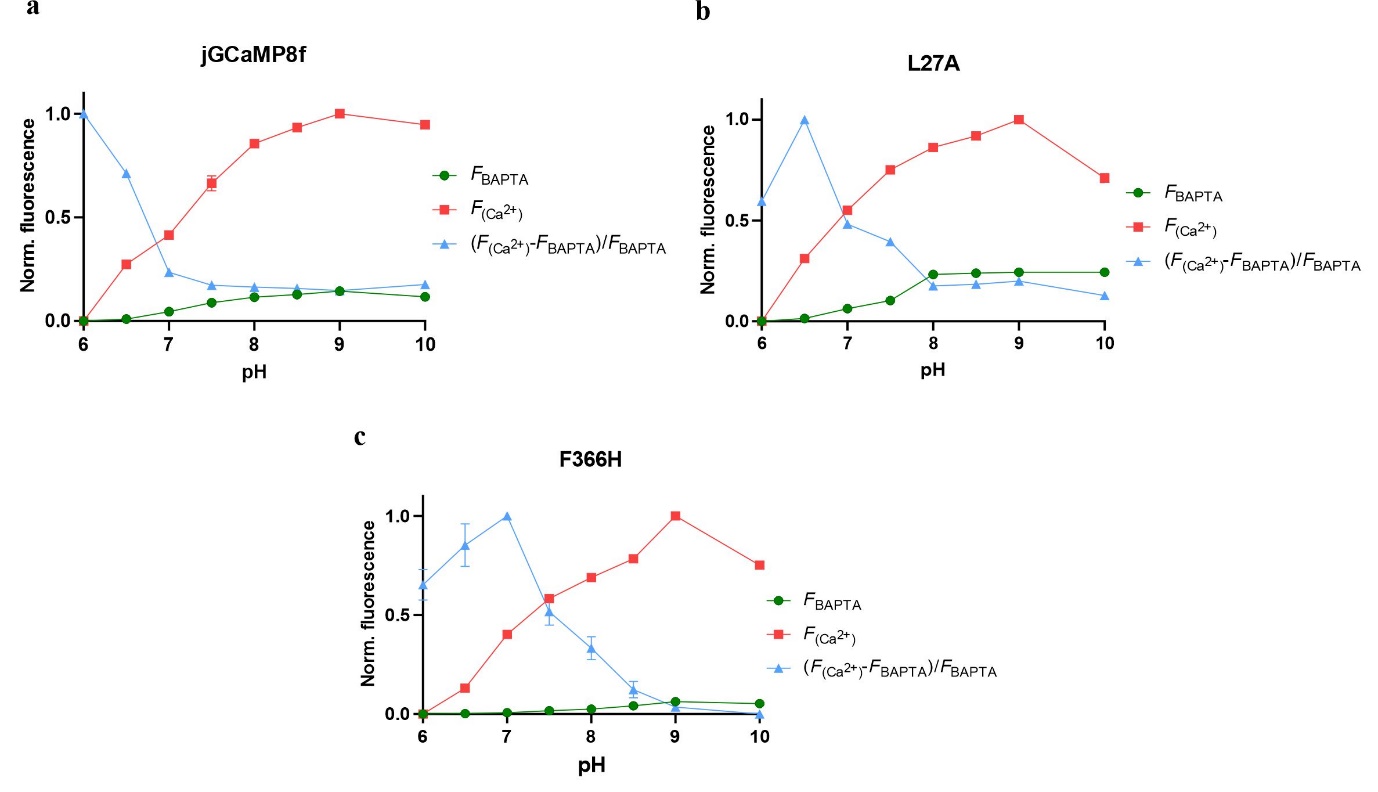


**Supplementary Figure 1**. pH sensitivity and p*K*a determination of (**a**) jGCaMP8f; (**b**) jGCaMP8f F366H. Normalised fluorescence in the presence of 1 mM Ca^2+^ (■) and in 2 mM BAPTA (●); Δ*F*/*F*_0_ (▲).


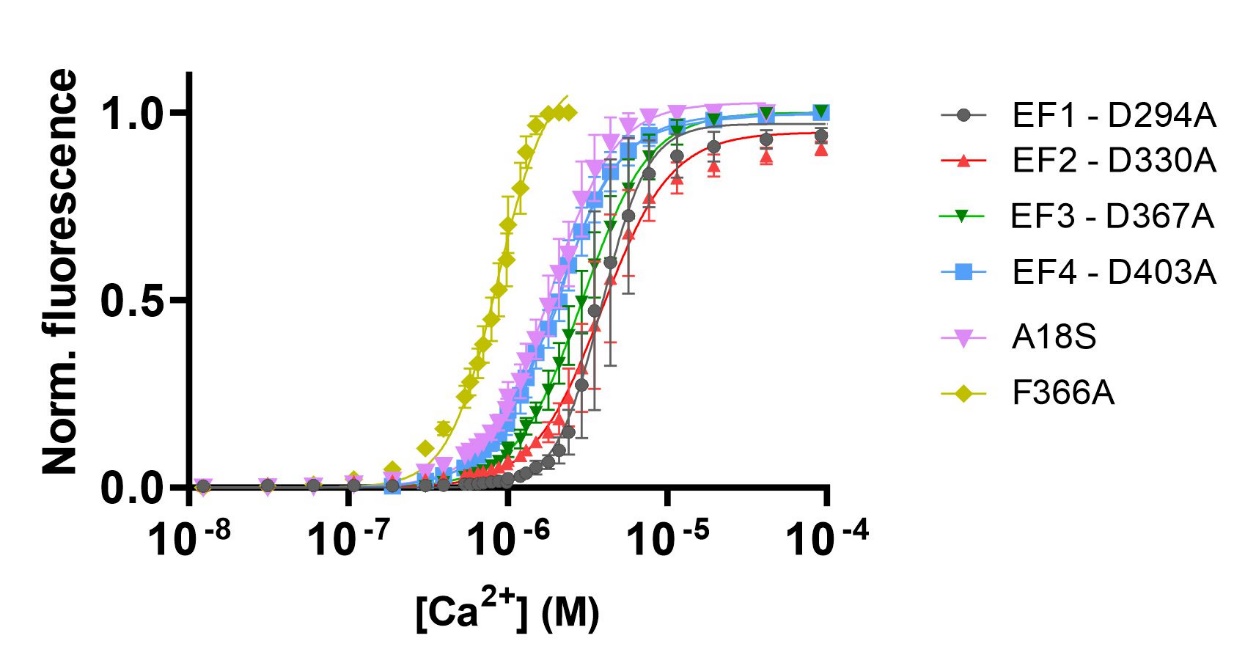


**Supplementary Figure 2**. Equilibrium titration curves of other GCaMP8f variants. Fitted parameters are displayed in **Table 1** and **Suppl. Table 1**.

**Supplementary Table 1**. Brightness measurements of jGCaMP8f and its novel variants L27A and F366H.

*^a^*Zhang et al., 2023.

| Variant | **jGCaMP8f** | | **L27A** | | **F366H** | |
| --- | --- | --- | --- | --- | --- | --- |
|  | − **Ca^2+^** | **+ Ca^2+^** | **− Ca^2+^** | **+ Ca^2+^** | **− Ca^2+^** | **+ Ca^2+^** |
| *ε*_o(492 nm)_  M^-1^cm^-1^ | 8413 (+Mg^2+^)  *^a^*1930 (−Mg^2+^) | 54226 (+Mg^2+^)  *^a^*50800 (−Mg^2+^) | 7726 (+Mg^2+^) | 48107 (+Mg^2+^) | 905 (+Mg^2+^) | 21203 (+Mg^2+^) |
| *φ* | 0.44 (+Mg^2+^)  *^a^*0.64 (−Mg^2+^) | 0.52 (+Mg^2+^)  *^a^*0.49 (−Mg^2+^) | 0.51 (+Mg^2+^) | 0.54 (+Mg^2+^) | 0.33 (+Mg^2+^) | 0.53 (+Mg^2+^) |
| *Brightness*  mM^-1^cm^-1^ | 3.7 (+Mg^2+^)  N.D. (−Mg^2+^) | 28.3 (+Mg^2+^)  N.D. (−Mg^2+^) | 3.95 (+Mg^2+^) | 25.9 (+Mg^2+^) | 0.3 (+Mg^2+^) | 11.3 (+Mg^2+^) |
| *Dynamic range* | 1 (+Mg^2+^) | 7.7 (+Mg^2+^) | 1 | 6.6 (+Mg^2+^) | 1 (+Mg^2+^) | 37.9 (+Mg^2+^) |

**Supplementary Table 2.** Summary data for other jGCaMP8f variants

^a^See **Figure 6**. ^b^The fast initial *on*-rate only gains prominence in amplitude at [Ca^2+^] > 20 μM.


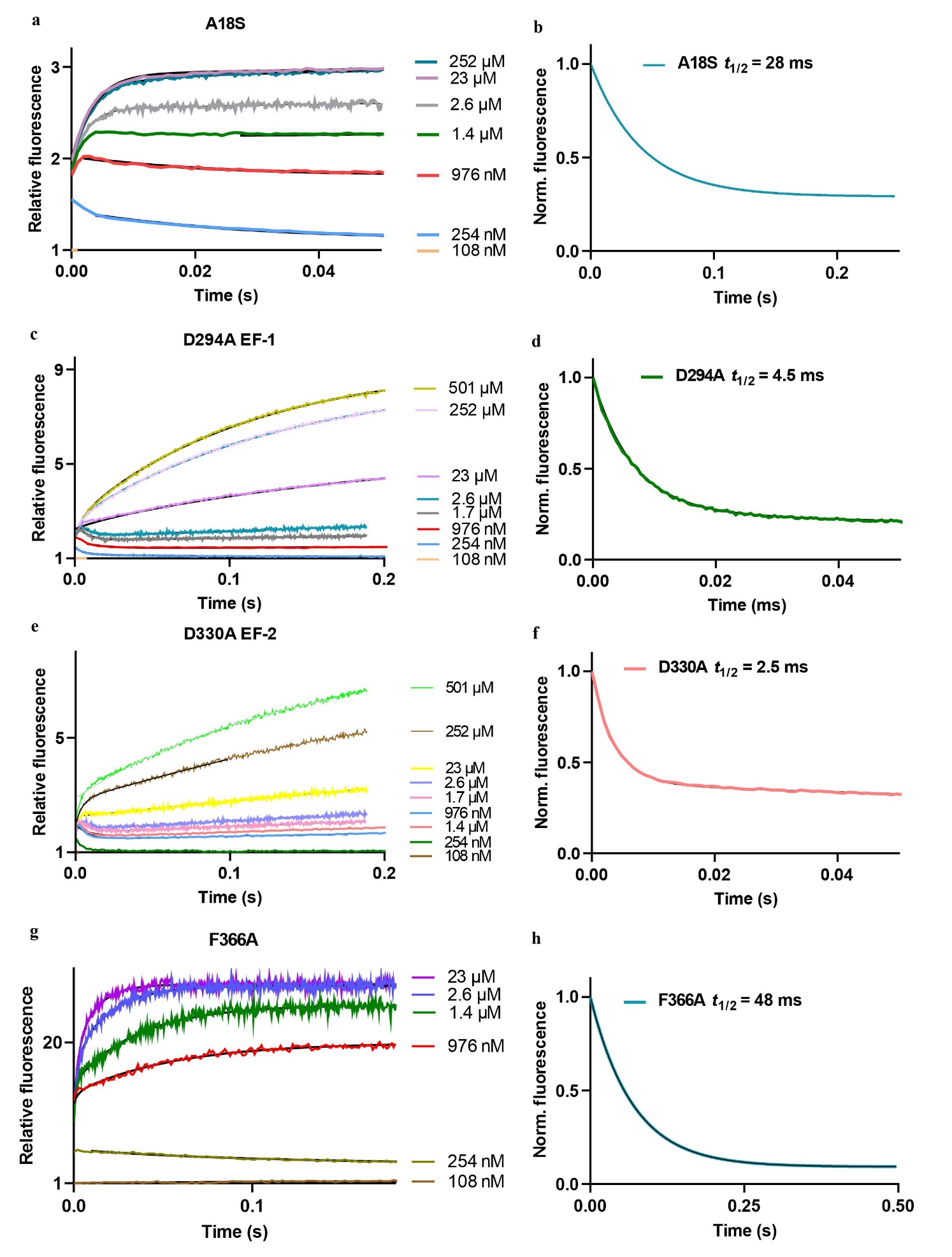


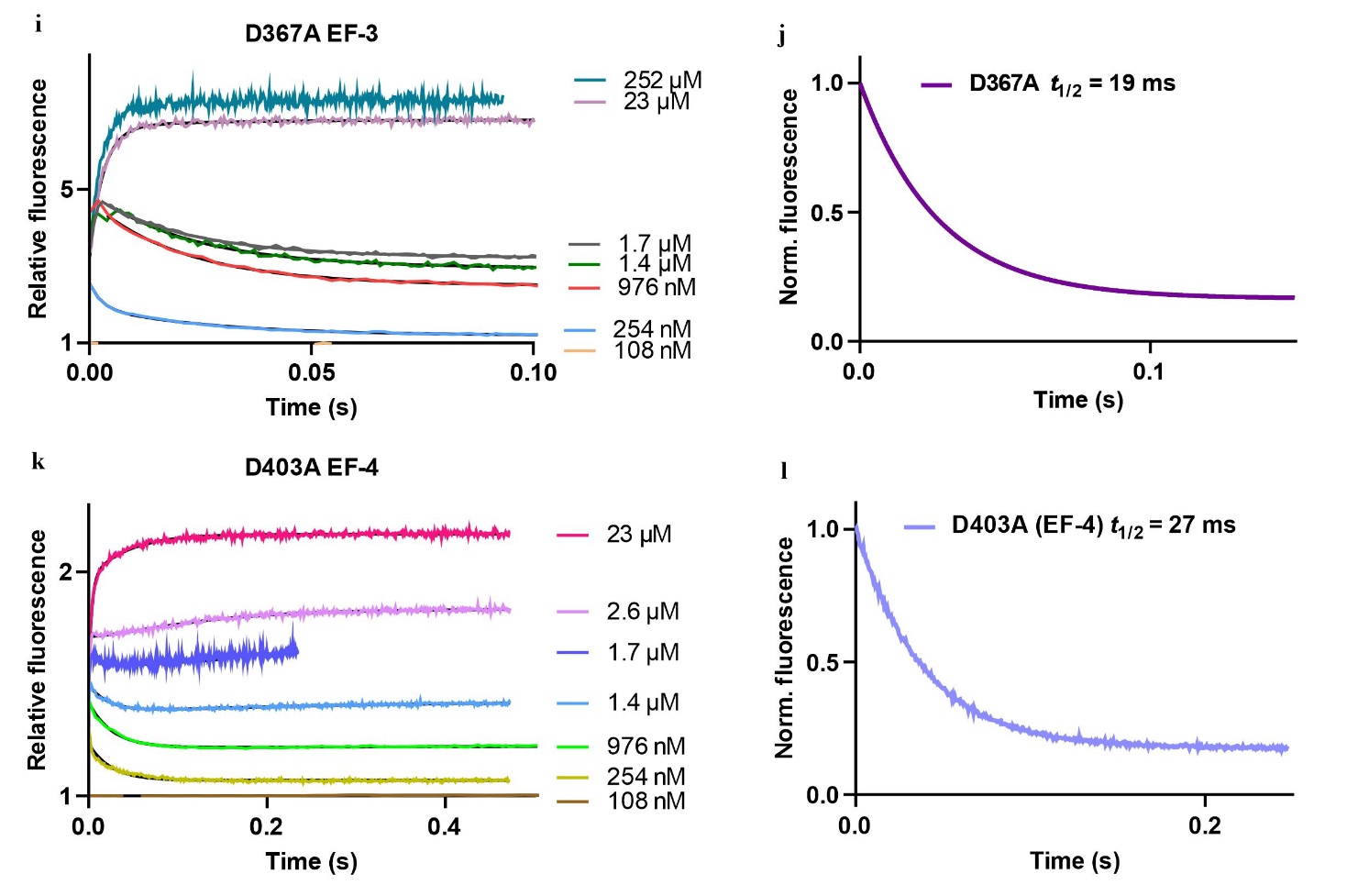


**Supplementary Figure 3**. Association (left panel) and dissociation (right panel) kinetic records for other jGCaMP8f variants. (**a**,**b**) A18S, (**c,d**) D294A (EF-1), (**e,f**) D330A (EF-2), (**g,h**) F366A, (**i,j**) D367A (EF-3) and (**k,l**) D403-A (EF-4).
